# Infrared Metasurface with a Top Cross-Square Nanobrick Array for Realizing a Highly Efficient Lightwave Absorption Across a Broad Wavelength Region

**DOI:** 10.3390/ma19061114

**Published:** 2026-03-13

**Authors:** Han Chen, Wuyang Ji, Chuang Zhang, Xuan Shao, Xinzhe Yao, Fangchen You, Haiwei Wang, Xinyu Zhang

**Affiliations:** 1 National Key Laboratory of Science & Technology on Multispectral Information Processing, Huazhong University of Science & Technology, Wuhan 430074, China; 13278854813@163.com (H.C.);; 2 School of Artificial Intelligence and Automation, Huazhong University of Science & Technology, Wuhan 430074, China; 3 Wuhan National Laboratory for Optoelectronics, Huazhong University of Science & Technology, Wuhan 430074, China

**Keywords:** IR metasurface, strong radiation absorption, surface plasmons, electromagnetic shielding action, near-field lightwave excitation

## Abstract

A type of metasurface with a top cross-square nanobrick (CSNB) array is proposed for realizing a highly efficient infrared (IR) radiation absorption across a broad wavelength region covering three traditional atmospheric windows. The metasurface is successfully constructed by integrating a layered CSNB array over a composite dielectric bottom supported by a common silicon substrate. The metasurface sample experimentally exhibits an average radiation absorptivity of more than 86% and a very low transmittance of less than 2% in the 1.28–14 μm wavelength region measured. A polarized absorption sensibility of the incident lightwaves and an average IR absorptivity of more than 80% with an oblique incidence at 40° are also demonstrated. The strong broad IR absorption with a negligible radiation transmission can be attributed to the existence of an obvious electromagnetic shielding action of the nanocavity formed between adjacent titanium films, and further, the near-field lightwave excitation upon the CSNBs of the metasurface charged by incident lightwaves satisfying the resonant condition needed.

## 1. Introduction

As is known, infrared (IR) lightwaves cover a very broad wavelength region from 0.76 to about 200 μm or longer [1]. And three IR atmospheric windows [2] already cover several typical wavebands, including the night vision IR band (0.76–1 μm), the short-wave IR region (1–3 μm), the midwave IR region (3–5 μm), and the longwave IR region (8–14 μm) [3,4,5,6]. In some particular applications, both the IR absorption bandwidth and efficiency are two crucial indicators for evaluating the performance of the functional IR materials or structures [7,8]. In effectively capturing IR light energy, a long-term target is to continuously broaden the IR responding wavelength range and the absorbing efficiency. As shown, metamaterials, as a type of artificially synthesized material having some characteristics, for instance, a highly sensitive and enhanced incident radiation response, and presenting a negative refractive index [9], unable to be discovered in the natural world, were proposed early [10,11,12]. Currently, the metasurface as a type of two-dimensional metamaterial [13] presents some completely different physical properties and the action mechanism compared to conventional macroscopic natural materials. They are usually composed of numerous sub-wavelength micro-nano-architectures arranged periodically. At present, the basic research and application reveal that the surface plasmon resonance [14,15,16,17,18] excited over the metasurface should be a dominant factor to determine the interaction behavior with incident lightwaves in a very broad electromagnetic wavelength region. The surface electromagnetic wavefields outfrom the oscillated surface net charge clusters, including negative electrons and positive holes, according to a fundamental dipole moment mode, which can be viewed as a basic dipole antenna, demonstrate a patterned near-field distribution around the transient plasmonic metasurface stimulated by incident radiation already satisfying the wavelength resonant condition with a needed bandwidth. Generally, the spatial extension range away from the metasurface should be in a range from several tens of nanometers to several hundreds of microns, as the wavelength of incident lightwaves increases. Considering the case that common metallic materials can be used to provide a large amount of surface “free electron”, the patterned metal-dielectric interface has been widely utilized to respond to the external electromagnetic photons, exhibiting a strong surface plasmon generation and still short distance propagation along a polarized direction beyond the traditional lightwave diffraction limit [19,20,21]. These characters represent a promising advance of the ultra-compact optical and/or optoelectronic elements and functional micro-systems.

So far, optical metasurfaces have become a type of powerful means for controlling near-field IR reflection and absorption. As reviewed in the roadmap by Kuznetsov et al. [22], the research field has advanced rapidly across the material design and device applications. Generally, conventional plasmonic metasurfaces can be used to realize a strong wavefield localization but suffer restriction from a relatively high ohmic loss, while all dielectric metasurfaces enable a low-loss and also a high quality factor resonances, as exemplified by Yang et al. [23]. However, the typical single-component architectures will exhibit an inherent trade-off among the loss, the bandwidth, and the resonance strength. As shown, the hybrid metal–dielectric metasurface can be utilized to resolve the limitation by exploiting the multipolar coupling effect, as indicated by Guo et al. [24]. The configurations can be easily compatible with the scalable nanofabrication techniques, including nano-imprint lithography [25]. For mid-IR optical components, the hybrid designs can further broaden the operating waveband and, thus, improve the performance [26]. Therefore, a hybrid metasurface scheme is well motivated to achieve a balanced broadband absorption with a relatively low loss in the near-IR region.

A type of IR metasurface architecture with a top cross-square nanobrick (CSNB) array for highly efficient absorbing lightwaves in a broad wavelength region is proposed in this article. The modeling and simulations based on common Lumerical FDTD are carried out. Firstly, the basic structural design and then parameter optimization concerning several factors such as the dielectric layer configuration and the CSNB construction, leading to an IR metasurface suitable for a broad wavelength range of 1~14 μm, are conducted carefully. By gradually increasing the number of layered CSNB for improving the IR responding and manipulating performances of the metasurface, an ideal structural configuration with the best IR absorption presented by an average IR absorptivity of more than 89% in the 1–14 μm wavelength region, is obtained. The mechanism of the metasurface architecture demonstrates that an ideal IR absorption efficiency is revealed mainly based on the construction of the nearfield lightwave upon the CSNB array of the metasurface charged by incident IR lightwaves with the needed wavelength, finally.

## 2. Principle and Simulation

**Figure 1:**
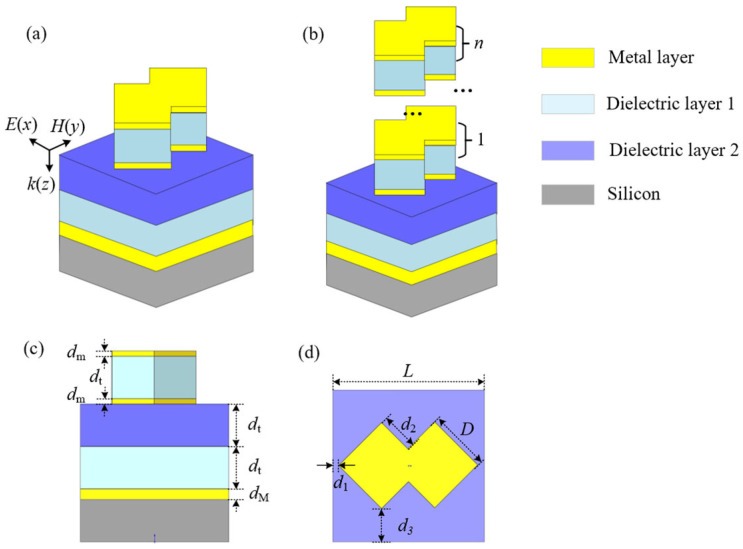
A basic CSNB over a layered dielectric film system with needed thickness configuration. (**a**) A 3D viewing of an element metasurface architecture configurated by a basic CSNB; (**b**) A top viewing of the overlapped CSNBs upon a composite dielectric element constructed; (**c**) The main film system configuration with needed thickness; (**d**) A top viewing of the metasurface element attached by several basic structural parameters.

A type of metasurface for highly efficient responding IR radiation is constructed by forming an arrayed CSNB upon a composite dielectric layer coated directly over a common silicon substrate, as shown in Figure 1. A basic CSNB is shaped through sandwiching a light blue dielectric layer-1 between the top and bottom yellow cross-square metal layers, which is symmetrically located over an element tailored segmentally from a composite dielectric layer constructed over the silicon substrate. A traditional rectangular coordinate system is labeled by a couple of electromagnetic components of *E*(*x*) and *H*(*y*) guided by a wave-vector *k*(*z*). Generally, a grey twin polished silicon wafer is selected as a substrate supporting a composite dielectric film system coated layer by layer over a yellow metallic film pre-fabricated upon one endface of the silicon substrate. And a light blue dielectric layer-1 and then a dark blue dielectric layer-2 are fabricated successively, as shown in Figure 1a. A single CSNB with a cross-square top is shaped by reasonably intersecting two nano-squares with the same dimensions. According to the design, the overlapped number of the basic CSNB can be expressed by *n* (1 ≤ *n* ≤ 5), currently, as shown in Figure 1b. So, a set of essential structural parameter configurations is as follows: the dielectric layer-1 and -2 having the same thickness *d*_t_, the top and bottom metal layer also having the same thickness *d*_m_, the thickness of the metal layer fabricated directly upon the silicon substrate being *d*_M_, as shown in Figure 1c. The main facial dimensions include a side length *L* of a dark blue square element and a couple of the side lengths *D* and *d*_2_ of a yellow cross-square, as shown by Figure 1d. The arranging periods of the CSNB are all *L* along the *x*- and *y*-directions. The minimum intervals between two adjacent CSNBs are 2*d*_1_ along the *y*-direction and 2*d*_3_ along the *x*-direction, respectively.

Considering a typical character of titanium material having a relatively low radiation reflectivity in several traditional IR atmospheric windows, both the top and bottom titanium layers with needed thickness are formed for obviously reducing the overall IR reflection of the final metasurface designed. To achieve a highly efficient response to incident radiations by the metasurface, the IR radiation away from the bottom titanium layer, which is directly connected to the silicon substrate, should be very weak and the thickness *d*_M_ greater than a skin depth compared to the radiation wavelength processed. In general, the skin depth can be expressed as $\delta =\sqrt{\frac{2}{\omega \mu \sigma }}$, where *ω* represents the angle frequency of the incident lightwave, and *σ* and *μ* are the electrical conductivity and magnetic permeability of the metal material used, respectively. According to the functioned configuration of the metasurface designed, an allowed maximum skin depth of the titanium layer must be more than 70 nm, the parameter *d*_M_ being about 100 nm.

The dielectric layer-1 is fabricated utilizing a common SiO_2_ material with excellent transparency and a low electromagnetic loss in the IR wavebands targeted. Generally, its high dielectric constant can be effectively used to excite relatively strong surface plasmons over the surface of a metal layer and further at the interface between the metal and dielectric layers, which indicates an obvious generation and/or enhancement of the propagating or localized surface wavefields, thereby leading to a significant improvement in the equivalent absorption (EA) of the metasurface across a wide electromagnetic waveband based on the near-field lightwave excitation. As demonstrated, the SiO_2_ material generally exhibits a better adhesion to common metal materials such as titanium, making it an ideal coupling under the mature micro-nano-fabrication flow, currently. The dielectric layer-2 is formed by common Si_3_N_4_ material, which usually demonstrates a high transparency and refractive index in traditional IR wavelength regions. So, it can also be utilized to stimulate strong travelling or localized surface wavefields. It can be expected that a highly efficient radiation manipulation can be conducted in an EA mode based on a strong nearfield lightwave generation and enhancement around the plasmonic metasurface.

Firstly, the metasurface architecture with a single layer of the CSNB array is constructed. Generally, the factors such as the physical property of the dielectric materials and their thickness, the titanium thickness and its nano-patterned morphology, will present a significant impact on the radiation processing efficiency of the metasurface formed. It should be noted that the simulations already regarded as an absorption of almost cold IR radiation, only based on a single CSNB, will result in an insufficient EA expression about the integrated metasurface architecture with an arrayed CSNB. Practically, a small existing deviation can be corrected easily. During the performance of the conventional FDTD simulations, the periodic boundary conditions are set in the *x*- and *y*-directions, respectively. A perfectly matched layer boundary condition is set only in the *z*-direction to sufficiently eliminate the boundary lightwave reflection and then obviously improve the simulation accuracy.

**Figure 2:**
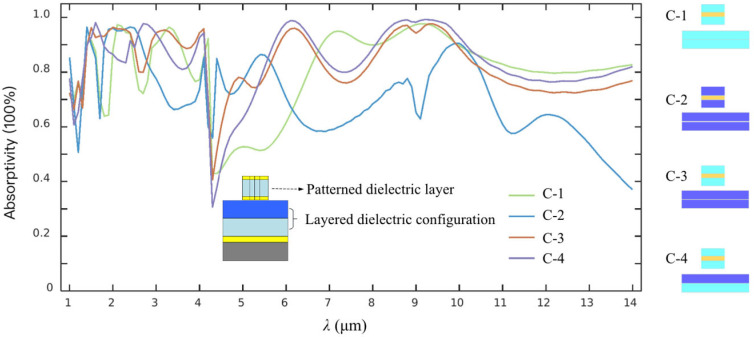
Equivalent IR absorption spectra of the metasurface architecture with different layered dielectric configuration labeled by C-1 to C-4.

As is known, a fundamental design consideration leading to an ideal functioning metasurface is to shape an arrayed micro-nano-texture surface of the composite architecture for a highly efficient response to the incident lightwaves by exciting the localized surface resonant wavefield array. So, the dielectric layers should be divided into two parts, including the top discontinuous element array and the bottom continuum, as illustrated in Figure 2. The typical four micro-nano-structure configurations include: (1) both the bottom continuum and the top discontinuous structural element being SiO_2_ labeled by C-1, (2) both the continuous and discontinuous structures being Si_3_N_4_ labeled by C-2, (3) the continuum being Si_3_N_4_ and the top discontinuous element being SiO_2_ labeled by C-3, (4) the continuum being Si_3_N_4_/SiO_2_ composite dielectric film system and the top element only being SiO_2_ labeled by C-4. Their typical spectral absorption behaviors in the IR waveband of 1~14 μm can be evaluated carefully according to a key parameter *A*(ω) for expressing the EA features discussed. It can be calculated by a simple relation of *A*(ω) = 1 − *R*(ω) − *T*(ω), where *R*(ω) and *T*(ω) are the reflectivity and transmittivity of the metasurface, and ω is the angular frequency of the incident radiation. It can be viewed that the spectral EA of the metasurface structures labeled by C-1, C-3 and C-4, demonstrate a similar wave-shaped variance trend with an average EA of about 82% in the wavelength range mentioned, which are slightly different with those indicated by C-3 already presenting an obviously lower average EA and also a rapid dropping after the radiation wavelength being more than about 12.14 μm. Noting that an intensive intrinsic IR absorption of Si_3_N_4_ material can be observed indirectly at the wavelength of about 4.32 μm, resulting in an extremely weak incident radiation response of the metasurface constructed.

The detailed average EA data in the different wavebands selected are given in Table 1. As shown, the average EA values simulated mainly based on SiO_2_ dielectric material are all more than about 80%, but those based on Si_3_N_4_ material are only about 70% in the entire wavelength region of 1–14 μm. In general, the EA data of the metasurface structures labeled by C-1 to C-4 all present a slight fluctuation around an essential value of about 80% in three traditional atmospheric windows. It should be noted that SiO_2_ material plays a crucial role in presenting a relatively strong equivalent radiation absorption in the IR region discussed, thanks to its ability to make an equivalent impedance closer to the background air, thereby achieving a stronger nearfield lightwave generation and further enhancement. It can be observed that the overall average EA of that labeled by C-4 is better than that of C-1 to C-3. So, a layered dielectric design by forming an arrayed discontinuous CSNB or a patterned SiO_2_ film system separated by a thin titanium layer over a Si_3_N_4_/SiO_2_ continuum to shape the metasurface labeled by C-4, is ultimately adopted for fabricating the metasurface sample.

**Figure 3:**
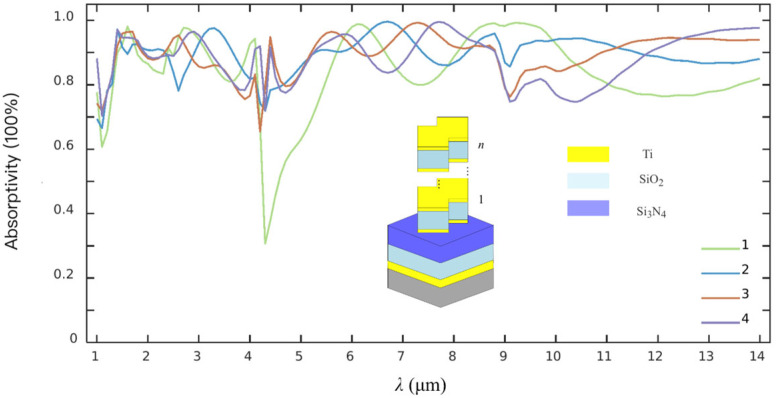
Typical EA spectra of the metasurface architectures with different *n* of the CSNB.

According to the simulations above, the factor that the average EA value of the metasurface is less than about 83% should be attributed to a relatively lower equivalent absorbing efficiency in the waveband of 3–5 μm. An operation for improving the EA in the wavelength range of 1–14 μm has been conducted by increasing the number of the CSNB vertically. Considering the case that the spacing between adjacent elements and their height usually depend on the achieved precision of micro-nano-fabrication, an optimized processing is performed to determine the final dielectric layer number n and the structural parameters. The typical EA spectra of the metasurface architectures with different n from 1 to 4 are shown in Figure 3. It can be observed that the average EA is apparently improved from less than about 83% of only having a single layer of CSNB to more than 88%. It should be noted that an intrinsic absorption valley at about 4.2 μm wavelength is completely removed after increasing n from the initial value 1. Almost the same EA of more than 89% corresponding to *n* being 2 or 3 indicates that the absorption stability of the double- or three-layered metasurface structure is significantly better than other structures, which also means that a rational *n* should be 2 because of a low-cost fabrication and basically the same radiation absorption performance.

The simulated average EA of the four metasurface architectures in each waveband is presented in Table 2. The average EA of more than about 86% in three wavebands and about 89% in the entire wavelength range highlights a relatively strong IR absorption stability of the double- and three-layered composite architectures.

**Figure 4:**
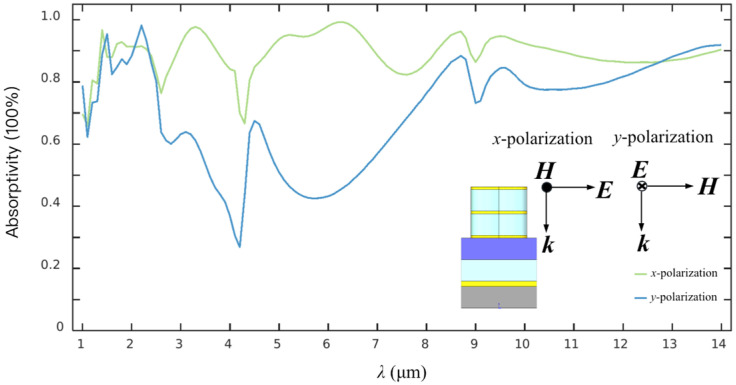
Typical EA spectra of the metasurface with 2-layered CSNB configuration along *x*- and *y*-polarization orientation, respectively.

The polarized EA characteristics of the metasurface architecture with 2-layered CSNB are further simulated, as shown in Figure 4. The IR radiations with two basic components along *x*- and *y*-polarization orientation, respectively, are vertically incident upon the surface of the metasurface. It can be viewed that both the spectral EA curves demonstrate an obvious difference, mainly in the 2.61~8.74 μm region, where an apparent drop with a minimum EA of about 26% along *y*-polarization orientation is remarkably weak to a relatively stable variance with an average EA of more than 89% along *x*-polarization orientation. The average EA of the metasurface architecture under *x*- and *y*-polarization orientation is more than about 89% and 75%, respectively.

As shown in Figure 4, the proposed metasurface exhibits an obvious polarization sensitivity, which should be attributed to the asymmetric structure design. As is known, the polarization sensitivity is not conducive to applications such as thermal energy harvesting and IR stealth, which require an unpolarized radiation response to present the important applications in IR polarized sensing, polarimetric imaging, target detection, and environmental monitoring. To address the polarization sensitivity issue, we select the rotationally symmetric or orthogonally arranged structures to achieve a 90° rotational symmetry in subsequent optimization. During the fabrication, the multilayer films are stacked consecutively, and then patterned by a single-step lithography and etching, which means that no inter-layer alignment is required during the manufacturing process. Compared with the single-layer metasurface, the fabrication complexity is almost at the same level, showing a good simplicity and scalability.

**Figure 5:**
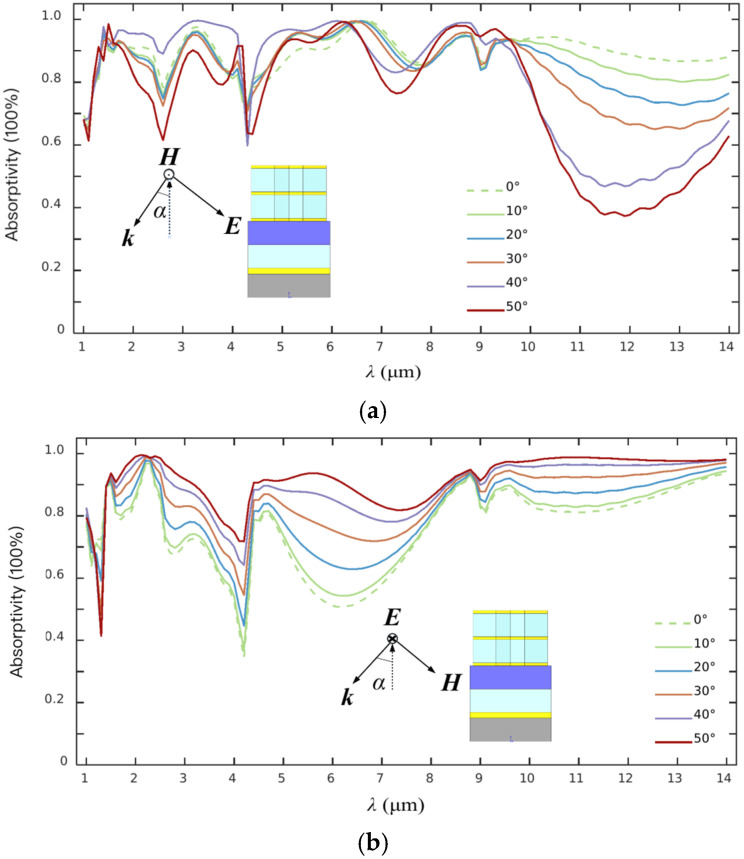
Spectral EA characteristics of the metasurface at different α along (**a**) *x*- and (**b**) *y*-polarization orientation.

Generally, the incident direction of IR lightwaves upon the metasurface, which can be expressed by an incident angle α between the incident direction and the normal direction, will also affect its EA characteristics. Usually, an ideal metasurface must present a high radiation absorption performance and EA stability in a wide range of incident angles. The typical EA spectra corresponding to different α including 0°, 10°, 20°, 30°, 40°, and 50°, are demonstrated in Figure 5. 

The incident radiation polarized along *x*-orientation already exhibits a gradually weakened wave-shaped variance trend, which is mainly represented in the 9.5–14 μm waveband, as shown in Figure 5a. Noting that the absorption valleys do not change their spectral location with the change in the incident angle.

The situation about the radiation absorption upon α in the same variance range only along *y*-polarization orientation is shown in Figure 5b. The obvious variance about the EA character can be clearly viewed in the waveband of 2.5~9 μm, mainly, which are apparently different from those polarized along the *x*-direction. As the incident angle increases, the wave-shaped EA spectra are gradually enhanced according to the EA variance behaviors.

As α being 0°, the IR absorption obviously presents four absorbing valleys at about 1.32 μm, 4.35 μm, 7.36 μm, and 11.52 μm, respectively. A similar character, where the absorption valley position does not change with the incident angle, can also be observed. The detailed EA data corresponding to the polarized incidence variance are listed in Table 3. As indicated, the average EA under *x*- and *y*-polarization will be decreased from an initial value of about 89% at 0° to about 74% at 50°, and then increased from the initial value of about 75% at 0° to about 91% at 50°, respectively. Although the radiation absorption efficiency under *y*-polarization with a small incident angle is slightly bad to that under *x*-polarization, it is more suitable to performing radiation absorption with a large incident angle in the long wavelength region.

**Figure 6:**
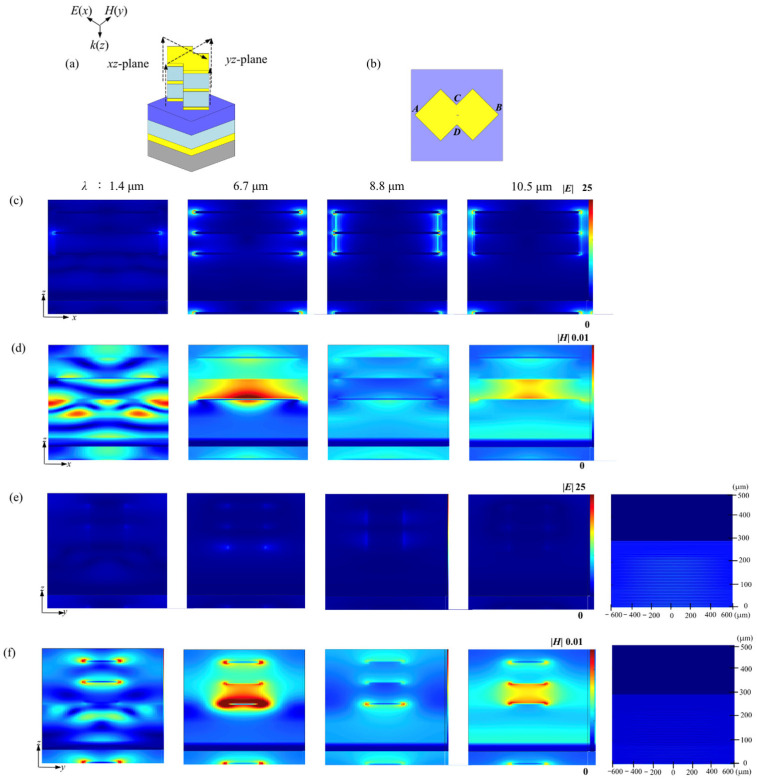
Spatial electric- and magnetic-field distribution over *xz*- and *yz*-plane, which are excited by IR radiation in a vertical incidence and attached by two spatial electric- and magnetic-field extending simulation in a nearfield fashion. (**a**) Schematic diagram of the basic CSNB over a composite dielectric bottom; (**b**) A top viewing of an element metasurface involving a basic top cross-square pattern; (**c**,**e**) Typical electric-field distribution over *xz*- and *yz*-planes at several wavelength points; (**d**,**f**) Typical magnetic-field distribution over *xz*- and *yz*-planes at the same wavelength points mentioned.

The typical spatial electric- and magnetic-field distribution over both the *xz*- and *yz*-planes at several featured wavelengths of 1.4 μm, 6.7 μm, 8.8 μm, and 9.5 μm, is exhibited in Figure 6. A basic structural configuration and a top cross-square pattern are demonstrated in Figure 6a,b. The typical transient electric-fields are presented in Figure 6c,e, and the corelated magnetic-fields in Figure 6d and f, respectively. A pair of the negative and positive net charge clusters accumulated over two opposite tips of three patterned titanium masks in a single 2-layered CSNB along the *x*-direction can also be presented, which can be viewed as a typical molecule antenna with a nanometer-scaled dipole moment length. It should be noted that two nanocavities have been architectured by coupling three cross-square titanium layers. As shown, a slight amplitude variance around each opposite tip of a titanium mask can be observed at different wavelengths mentioned above. Usually, the net charge distribution density at each titanium tip should be correlated closely with the amplitude (intensity) of the electric-fields around a single molecule antenna, as illustrated by each bright point according to a color bar attached. A transient net charge distribution over the same side of the adjacent titanium masks, for instance, presents a sequence of positive–negative–positive at one side and another sequence of negative–positive–negative at the opposite side. So, two adjacent brightest spots over two titanium masks already connected by a short bright line, are exhibited at the wavelength of 8.8 μm. The weakened spatial electric-field distribution can also be observed at two typical wavelength points of about 6.7 μm and about 10.5 μm. Consequently, two semi-opened nanocavities filled fully by dielectric films are architectured by closely coupling both the top and intermediate titanium layers and further the bottom and the same intermediate titanium layers, respectively. According to the indication of the color bar, an obviously weakened electric-field shielding action within two nanocavities can be observed, as presented by Figure 6c. And a weaker spatial electric-field distribution over the *yz*-plane can also be observed in Figure 6e. The correlated spatial magnetic-field distributions can be viewed in Figure 6d,f.

So, a total lightwave shielding of the metasurface must involve two contributions of both the spatial electric and magnetic fields. It should be noted that a strong radiation absorption of the metasurface should be governed by the physical mechanism that nearfield-typed standing-waves upon the top CSNB are formed by a strong spatial interference between incident lightwaves and the re-emitted nearfield lightwaves from densely distributed molecule antennas based on both the surface plasmon polaritons (SPPs) outfrom the net charge re-arrangement over the bottom titanium film, and the localized surface plasmas (LSPs) of a couple of the positive and negative net charges accumulated and then attenuated over two tips of each titanium mask. As shown, a relatively strong magnetic-field will be sealed in the second nanocavity below the top nanocavity. Noting that the patterned electromagnetic wavefield distribution formed upon the metasurface indicates that the frequency and intensity of incident radiation already match the resonant condition. So, the incident IR energy will be highly localized within the nearfield region and then decay rapidly as the distance away from the metasurface gradually increases, thus completely vanishes after the viewing distance is more than about 280 μm, as indicated by simulations.

As demonstrated, impedance matching is a key physical mechanism for achieving an efficient broadband absorption. When an effective impedance of the metasurface is matched with that of free space, the IR reflection at the interface between the metasurface and free space can be maximally suppressed, and thus the incident radiation efficiently enters the interior of the metasurface almost without loss, due to a strong interface reflection. Based on the field distribution characteristics shown in Figure 6, it can be noted that the incident radiation entering the interior of the metasurface will strongly interfere the SPPs generated by the rearrangement of the surface net charges on the bottom titanium film, as well as the near-field lightwaves re-emitted by the localized LSPs generated by the positive and negative net charges accumulated at the tips of the titanium mask. So, the near-field standing wave can be formed over the surface of the CSNB at the top, resulting in a highly localized incident energy in the near-field region of the metasurface.

A synergistic effect of the impedance matching and plasmonic interference will jointly support the broadband absorption of the metasurface. The impedance matching will ensure an efficient coupling of the incident lightwave and thus reduce the interface losses. And the synergistic effect between SPPs and LSPs will also promote an efficient absorption and then attenuation of incident energy inside the metasurface, which is highly consistent with the net charge distribution and also the electric- and magnetic-field distribution characters, as observed in Figure 6. So, the net charge accumulation at the tips of the titanium mask corresponding to the excitation of LSPs and the field strength distribution inside the nanocavity will enhance the formation of the standing wave fields and the energy localization, further verifying the rationality of the impedance matching analysis.

In addition, the variation in the relative impedance will show a good correspondence with the field strength distribution with different wavelengths of 1.4 μm, 6.7 μm, 8.8 μm, and 10.5 μm, as shown in Figure 6. At the wavelength of 8.8 μm, the relative impedance Z/Z_0_ is closest to 1, which means that the impedance matching is optimal. Correspondingly, at this wavelength shown in Figure 6, the titanium mask exhibits several factors, including the strongest field strength, the densest charge distribution, and the highest absorption efficiency. At the wavelengths of 6.7 μm and 10.5 μm, the real part of the relative impedance will slightly deviate from 1, and the absolute value of the imaginary part will be slightly increased, resulting in a slight decrease in impedance matching action. This is consistent with the phenomenon of the weakened field intensity distribution at the wavelength shown in Figure 6, to further support the decisive influence of impedance matching on absorption efficiency.

## 3. Fabrication and Evaluation

**Figure 7:**
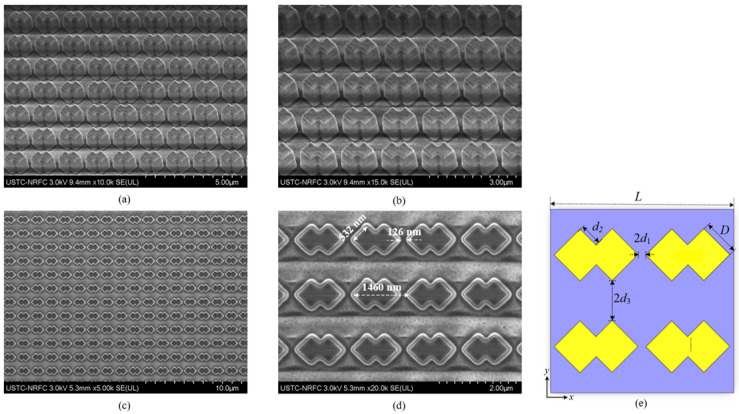
SEM photographs of the metasurface sample. The side viewing corresponding to the top CSNB array at a magnification of 10 k (**a**) and 15 k (**b**). The top view of the functional region at a magnification of 2.5 k (**c**) and 20 k (**d**). Top view of metasurface structure (partial) (**e**).

The metasurface samples are fabricated using mature micro-nano-fabrication techniques (Thermo Fisher Scientific, Milwaukee, WI, USA), including the conventional magnetron sputtering, PECVD, UV photolithography, and dry etching, as shown in Figure 7. Both the morphology and the main structural parameters of the metasurface samples are acquired utilizing the field emission scanning electron microscopy of Nova NanoSEM 450 (Thermo Fisher Scientific, Milwaukee, WI, USA). The 3D appearances of a partial metasurface structure with a clear layered arrangement are presented in Figure 7a,b, and two top views in Figure 7c,d. As shown, both the intersecting squares already present an angle of nearly 90°, and the distribution period of the cross-square-shaped nanostructure with a minimum spacing of 126 nm is 1460 nm. The side lengths of each nano-square at the top and bottom of a single CSNB are 532 nm and 604 nm, respectively. The measured data only present a small difference to the simulations, which means a manufacturing error of less than 15%. The main side lengths of the CSNBs are *D* = 565 nm, *d*_2_ = 280 nm, *d*_1_ = 90 nm, and *d*_3_ = 300 nm, respectively, as shown in Figure 7e. It should be noted that it is impossible to fully ensure the verticality of the sidewalls of a single CSNB during performing a multi-layer etching, as shown by the upper and lower bottom areas of the nano-structure being slightly smooth.

**Figure 8:**
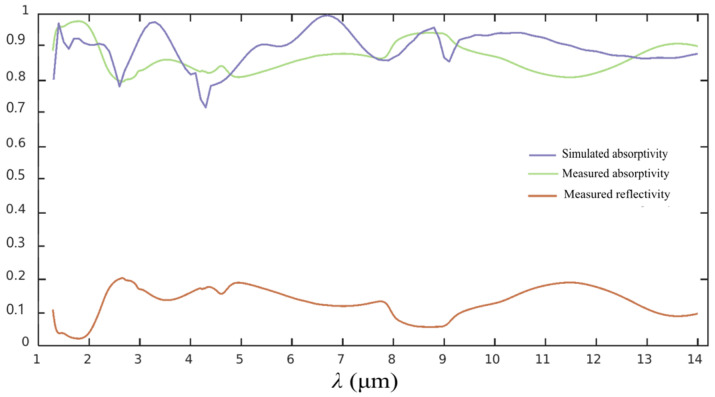
Measured and simulated EA spectra of the metasurface sample.

The background spectra are first collected using a Nicolet iN10 micro IR spectrometer (Thermo Fisher Scientific, Milwaukee, WI, USA). The metasurface sample is placed on the stage, which is located at the testing area, through a microscope for collecting both the reflection and transmission spectra. The designed square nanostructure has a side length of 565 nm and a period of 1400 nm. The nanostructure sample has a side length of 532 nm and a period of 1460 nm, as shown in Figure 7d. As shown in Figure 8, the measured transmittance of the functional region is basically 0, which is consistent with the simulations, although very little short-wavelength IR radiation is reflected from the structural region. The spectral variance trend of the EA is basically consistent with the simulation, but more smoothly, which can be viewed as a spectral guidance. The metasurface sample basically maintains an overall average EA of more than 86% in the measured wavelength range of 1.28–14 μm. The detailed EA data in several typical wavebands are listed in Table 4. The average EA with a relatively large value indicates that the sample presents an extremely excellent radiation absorption performance in the short waveband of 1.28–3 μm. In the waveband of 3–8 μm, the EA demonstrates a relatively steady spectral variance without an obvious absorption peak or valley. In the long waveband of 8–14 μm, the sample presents an EA of more than 87%. So, the EA data indicate that the prepared metasurface sample has presented an ideal IR absorption performance in an ultra-wide spectral region of 1.28–14 μm.

From Table 5, it can be seen that the height of the designed structure is the lowest, and the bandwidth is the highest. Although the absorption rate is not as good as others, the difference is not significant. The structures involved are considered to be good broadband absorbers in terms of overall performance.

As observed in Figure 8, the simulations and measurements are generally consistent, but still show some slight discrepancies. Especially, the sharp resonance depressions appear at 4.3 μm and 9 μm. The first reason is that the optical constants of the prepared SiO_2_ and Si_3_N_4_ thin films differ from the standard database used in FDTD simulations. After updating the measured optical constants, the absorption spectra become smoother. The second reason should be attributed to remain a certain gap between the size and shape of the fabricated structure used in the simulations. As indicated, the employed photoresist exhibits a broadened appearance after lithography and development, leading to the deviation between the actual size and the designed value. Although an appropriate coating size is calculated after a series of tests in previous work, a more precise combination of the photoresist dosage and the patterned photoresist size should be adopted to realize an optimal parameter combination. On the other hand, the etching process cannot ensure that the nanostructure exhibits a perfectly vertical state. In future work, more etching parameters will be compared and optimized for selecting an optimal parameter set, thereby making the nanostructure present an ideal vertical appearance.

## 4. Conclusions

A type of IR metasurface with an arrayed top CSNB is proposed for performing a highly efficient radiation absorption across a broad wavelength region covering three traditional atmospheric windows. The metasurface is effectively constructed by configuring a 2-layered CSNB array over a composite dielectric bottom supported by a common silicon substrate. The metasurface sample experimentally exhibits an average radiation absorptivity of more than 86% and a very low transmittance of less than 2% in the 1.28–14 μm waveband measured. A polarized absorption sensibility of the incident radiation and an average IR absorptivity of more than 80% with an oblique incidence at 40° are demonstrated. The strong broad radiation absorption with a negligible light energy transmission can be attributed to the factor of the existing obvious electromagnetic shielding action of the nanocavity formed between adjacent titanium films, and further, the nearfield lightwave generated upon the CSNB array of the metasurface charged by incident lightwaves already satisfying the resonant condition needed.

## Figures and Tables

**Table 1:** Average EA data of the metasurface in several typical IR wavebands.

Layered Dielectric Configuration	Average EA in Different IR Waveband (%)			
	1~3 μm	3~5 μm	8~14 μm	1~14 μm
C-1	83.71	75.03	85.00	80.80
C-2	80.47	70.47	65.00	70.57
C-3	88.90	82.69	80.16	82.95
C-4	86.59	82.15	83.95	83.72

**Table 2:** Average EA data of the metasurface architectures with different *n* in the IR wavebands selected.

*n*	Average EA in Different IR Wavebands (%)			
	1~3 μm	3~5 μm	8~14 μm	1~14 μm
1	86.59	82.15	83.95	83.72
2	88.24	89.45	90.13	89.46
3	86.77	90.19	90.06	89.50
4	88.39	89.74	86.62	88.31

**Table 3:** Average EA data of the metasurface corresponding to different α along two polarized orientations.

Incident Angle *α*	*x*-Polarization (%)	*y*-Polarization (%)
0°	89.46	75.90
10°	87.98	77.36
20°	85.93	80.99
30°	83.60	85.37
40°	80.90	89.19
50°	74.83	91.79

**Table 4:** Typical EA data of the metasurface sample in several wavebands selected.

Waveband (μm)	Maximum EA (%)	Minimum EA (%)	Average EA (%)
1.28–14	97.85	80.02	86.93
1.28–3	97.85	80.02	89.43
3–8	90.78	81.00	85.15
8–14	94.33	80.94	87.48

**Table 5:** Comparison of different broadband absorbers.

Type of Absorbers	Bandwidth (μm)	Average EA (%)	Total Thickness (nm)
Layered CSNB	1–14	89.63	950
MIM structure [27]	1.1–1.7	88	1100
Multi-layer nanocone array [28]	0.5–5.7	98.2	1800
Metasurface coating [29]	3–13	85	1000
Reconfigurable honeycomb Structure [30]	1–4	90	1200

## Data Availability

The original contributions presented in the study are included in the article. Further inquiries can be directed to the corresponding author.
